# Association between Polymorphisms in the Promoter Regions of Matrix Metalloproteinases (MMPs) and Risk of Cancer Metastasis: A Meta-Analysis

**DOI:** 10.1371/journal.pone.0031251

**Published:** 2012-02-14

**Authors:** Dan Liu, Hong Guo, Yafei Li, Xueqing Xu, Kang Yang, Yun Bai

**Affiliations:** 1 Department of Medical Genetics, College of Basic Medical Sciences, Third Military Medical University, Chongqing, China; 2 Department of Epidemiology, College of Preventive Medicine, Third Military Medical University, Chongqing, China; 3 Department of Thoracic and Cardiac Surgery, Southwest Hospital, Third Military Medical University, Chongqing, China; Indiana University, United States of America

## Abstract

**Background:**

A variety of studies have evaluated the associations between polymorphisms in the promoter regions of Matrix metalloproteinases (MMPs) and cancer metastasis. However, the results remain inconclusive. To better understand the roles of MMP polymorphisms in metastasis, we conducted a comprehensive meta-analysis.

**Methods:**

Electronic databases were searched (from January 2000 to June 2011) for any MMP genetic association studies in metastasis. Overall and subgroup analyses were performed. Odds ratio (OR) and 95% confidence interval (CI) were used to evaluate the associations between MMP polymorphisms and metastasis. Statistical analysis was performed with Review Manager 5.0 and STATA11.0.

**Results:**

Thirty-three studies addressing five MMP polymorphisms were analyzed among 10,516 cancer cases (4,059 metastasis-positive cases and 6,457 metastasis-negative cases). For *MMP1 (−1607)1G/2G*, genotype *2G/2G* increased the overall risk of metastasis under the recessive model (OR = 1.44, 95% CI = 1.05–1.98). In subgroup analysis based on cancer type, associations were found in head/neck and breast cancer under the recessive model, and also in breast cancer under the dominant model. For *MMP3 (−1171) 5A/6A*, the polymorphism decreased the overall risk of metastasis under two genetic models (recessive: OR = 0.80, 95%CI = 0.64–0.99, dominant: OR = 0.72, 95%CI = 0.56–0.93). The polymorphisms of *MMP7 (−181) A/G* and *MMP9 (−1562) C/T* increased metastatic risk. However, no association was observed between *MMP2 (−1306) C/T* and metastasis.

**Conclusions:**

Our investigations demonstrate that polymorphisms in the promoter regions of *MMP1, 3, 7* and *9* might be associated with metastasis in some cancers. Further studies with large sample size for *MMP2* should be conducted.

## Introduction

The lethal outcome of the vast majority of cancers is due to the dissemination of metastatic tumor cells and the outgrowth of secondary tumors at distant sites. Several steps occur in cancer metastasis and invasion: dissociation of tumor cells at the primary site, local invasion, angiogenesis, intravasation into the vasculature or lymphatic systems, extravasation and proliferation at a distant site [1]. Metastasis and invasion require the crossing of several physical barriers such as the basement membrane or the adjacent connective tissue.

Matrix metalloproteinases (MMPs) are a family of zinc-dependent endopeptidases, which play critical roles in cancer progression and metastasis [1]–[2]. Based on the structure and substrate specificity, MMPs can be divided into five groups: collagenases, gelatinases, stromelysins, matrilysins and membrane MMPs [3]. MMPs are involved in normal physiological and pathological processes such as degradation and remolding of extracellular matrix, embryonic development, reproduction and cancer [4]–[5]. MMPs are the main group of proteolytic enzymes that are involved in cancer invasion and metastasis.

MMP1 and MMP3 are two important members in MMPs family. They are neighbors located on 11q22 and play important roles in cancer development and metastasis. MMP1 is one of the widely expressed MMPs that can degrade type I, II and III collagens. MMP3 is produced by connective tissue, which can activate other MMPs and release cell surface molecules. It can degrade numerous extracellular substrates, including collagens III and IV [6]. MMP2 is able to degrade type IV collagen and some bioactive molecules. Studies have shown that MMP2 is over-expressed in head and neck squamous cell carcinoma tissues with higher ability of invasion and metastasis [7]. MMP7 is a protease with broad substrate specificity, which can degrade elastin, fibronectin, and type IV collagen. It is the smallest member of MMP family and is over-expressed in many cancers. MMP9 is the most complex member of MMP family. It has proteolytic activity against type IV collagen, a major component of the basement membrane. The expression of MMP9 is upregulated in various human cancer types such as esophageal cancer, breast cancer and gastric cancer.

A variety of molecular epidemiological studies have focused on the associations between MMP polymorphisms and cancer susceptibility. Some functional single nucleotide polymorphisms, including *MMP1 (−1607)1G/2G* (rs1799750), *MMP2 (−1306) C/T* (rs243865), *MMP3 (−1171) 5A/5A* (rs3025058), *MMP7 (−181) A/G* (rs11568818) and *MMP9 (−1562) C/T* (rs3918242), have been identified [8]–[12]. McColgan's study [13] evaluated the associations between polymorphisms of *MMP1, 2, 3, 9* and susceptibility to lung, breast and colorectal cancers. MMP polymorphisms have been studied in cancer metastasis with disparate results, partly due to the small number of subjects in several studies. No meta-analysis has been conducted to reliably evaluate these associations so far. To better clarify the associations of these MMP polymorphisms with metastasis, we conducted a comprehensive meta-analysis by collecting and analyzing the published data.

## Materials and Methods

### Search strategy

Electronic databases of PubMed, ISI Web of Knowledge, Medline, Embase and Google Scholar Search were used to identify all published case-control studies that evaluate the associations between MMP polymorphisms and metastasis (between January 2000 and June 2011). The Medical Subject Headings and key words used for search were “metastasis”, and (“MMPs” or “matrix metalloproteinase”) and “polymorphism” and (“cancer” or “neoplasm”). The references of all identified publications were hand-searched for additional studies. Authors were contacted directly regarding crucial data not reported in original articles. Abstracts, unpublished reports and articles written in non-English languages were not included.

### Inclusion and exclusion criteria

The inclusion criteria were as follows: (1) independent case-control design was used to evaluate the association between MMP polymorphism and cancer in each study; (2) for each study, the score of quality evaluation was over 6 (Table S1); (3) the number or frequency of genotype was given in detail; (4) only genes with two or more studies on one polymorphism were included in our analysis.

The exclusion criteria were as follows: (1) studies with insufficient information were excluded, for example, genotype frequency or number not reported, or histopathological diagnosis of cancer not confirmed; (2) if the same population was included in previous studies, only the most recent or complete study was included after careful examination.

To minimize the bias and improve the reliability, two researchers extracted data with the inclusion and exclusion criteria independently and reached a consensus.

### Data extraction

Information such as the first author, publication year, country origin, cancer type, ethnicity of study population, genotyping method, number of metastasis-positive/negative cases and adjusting factors was collected from each study. For studies including subjects of different ethnicities, data were extracted separately and categorized as Asians and Europeans (Caucasians). If one study involved different cancer types, each cancer type was listed as a separate study.

According to the TNM classification standardizations, cancer patients were assigned to two subgroups named metastasis-positive and metastasis-negative based on the presence/absence of detectable lymph nodes or distant metastasis at the time of diagnosis or follow-up.

### Statistical analysis

Associations between MMP polymorphisms and metastasis were evaluated by odds ratio (OR) and 95% confidence interval (CI). In addition to overall comparison, we performed stratification analysis based on cancer type (if one type contained less than two individual studies, it was combined into the ‘other cancers’ group) and ethnicity of study population. Heterogeneity between studies was assessed using *Q* test and *p* and *I* ^2^ value. *I* ^2^ was a value that could describe the percentage of variation across studies, where 0–25% indicated no observed heterogeneity and larger values showed increasing heterogeneity, with 25–50% regarded as low, 50–75% as moderate, and 75–100% as high. *p*>0.05 for the *Q*-test indicated a lack of heterogeneity across studies, allowing to use the fixed-effects model (the Mantel-Haenszel method) [14]; otherwise, the random-effects model was used (the DerSimonian and Laird method) [15]. The heterogeneity was adjusted by subgroup analysis and meta-regression. The pooled ORs were performed on the dominant (BB+AB versus AA) and recessive model (BB versus AB+AA) respectively (A represented major allele, B represented minor allele). The significance of pooled ORs was tested by *Z* test (*p*<0.05 was considered significant). The funnel plot and Egger's test were used to examine the publication bias [16]. All *p* values were two-sided, and all statistical analyses were performed using Review Manager 5.0 and STATA11.0 software.

To ensure reliability and accuracy of the results, two researchers entered the data into the software program independently and reached a consensus.

## Results

### Study characteristics

**Figure 1:**
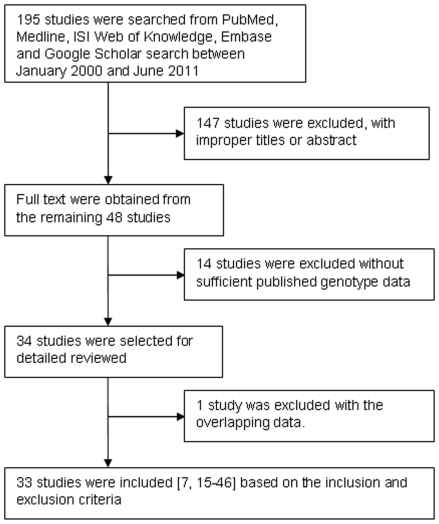
Flow diagram of study identification.

By the inclusion and exclusion criteria, 195 articles were found, but only 48 studies were preliminarily identified for further evaluation. After carefully evaluating the quality of the 48 remained articles, we excluded 15 studies, of which 1 study had overlapped data and 14 studies did not report detailed genotype data or genotype frequency information for metastasis-positive/negative cases. Finally, 33 relevant studies [7], [17]–[48] addressing five polymorphisms in five MMP genes analyzed in 10,516 cancer cases (4,059 metastasis-positive and 6,457 metastasis-negative cases) were included (Flow diagram shown in Figure 1). The study was judged to be of good quality if the total score was over 6, otherwise, of poor quality. The total score of most studies was over 6 except for four studies [28]–[29], [31], [33] (Table S2). The information of healthy controls was not provided in the four studies. However, we only focused on the associations of MMP polymorphisms with cancer metastasis, thus including the four studies.

Information including cancer type, publication year, country, ethnicity, genotyping method, genotype data, average age of cases and controls, sample size (case/control), Hardy-Weinberg equilibrium of controls, adjusting factors, determination of cancer and metastasis positive or negative group was listed in Table 1 and Table S3. There were 17 articles including 1,218 metastasis-positive and 1,337 metastasis-negative cases for *MMP1 (−1607) 1G/2G*, 4 articles with 2,234 cancer cases for *MMP2 (−1306) C/T*, 8 articles with 2,367 cancer cases including 783 metastasis-positive and 1,584 metastasis-negative cases for *MMP3 (−1171) 5A/6A*, 3 articles with 808 cancer cases for *MMP7 (−181) A/G* and 10 articles involving 2,552 cancer cases (1,129 metastasis-positive and 1,423 metastasis-negative cases) for *MMP9 (−1562) C/T*.

**Table 1:** Comparison of genotype distribution of MMP polymorphisms between cancer metastasis positive and negative subjects.

Gene	Cancer type	Country	Ethnicity	Metastasis(+)				Metastasis(−)			
				*N*	AA	AB	BB	*N*	AA	AB	BB
**MMP1 (−1607) 1G/2G**											
Cao 2005	head/neck	China	Asian	67	27^a^		40	29	14^a^		15
Hashimoto 2004	head/neck	Japan	Asian	43	20^a^		23	86	40^a^		46
Kondo 2005	head/neck	Japan/Taiwan	Asian	40	6		34^b^	43	4		39^b^
Nasr 2007	head/neck	Tunisia	European	118	5	37	76	56	8	26	22
O-charoenrat 2006	head/neck	Thailand	Asian	181	75^a^		106	119	76^a^		43
Shimizu 2008	head/neck	Japan	Asian	19	9^a^		10	50	23^a^		27
Kouhkan 2008	colorectal	Iran	European	69	31^a^		38	81	60^a^		21
Ghilardi 2001	colorectal	Italy	European	17	6^a^		11	43	31^a^		12
Woo 2006	colorectal	Korea	Asian	79	2	23	54	106	5	31	70
Jin 2005	gastric	China	Asian	46	2	16	28	48	7	16	25
Matsumura 2004	gastric	Japan	Asian	89	11	42	36	126	15	46	65
Hughes 2007	breast	London	European	52	12	20	20	88	26	43	19
Przybylowska2006	breast	Poland	European	141	33	57	51	129	44	58	27
Fang 2005	NSCLC^c^	China	Asian	123	13	41	69	74	8	24	42
Fong 2004	chondrosarcoma	Taiwan	Asian	14	6	8	0	53	12	26	15
Jin 2005	ESCC^d^	China	Asian	59	6	24	29	72	12	29	31
Lai 2005	cervical	Taiwan	Asian	51	12	22	17	89	8	38	43
Albayrak 2007	prostate	Turkey	European	10	3		7^b^	45	7		38^b^
**MMP2 (−1306) C/T**											
Cotignola 2007	melanoma	USA	European	129	86	39	4	866	543	281	42
O-charoenrat 2006	head/neck	Thailand	Asian	152	140		12^b^	87	66		21^b^
Lei2007	breast	Sweden	European	230	121	86	23	559	317	203	39
Wu2007	gastric	Taiwan	Asian	93	83	7	3	118	88	26	4
**MMP3 (−1171) 5A/6A**											
Hughes 2007	breast	London	European	50	16	29	5	85	23	44	18
Ghilardi 2002	breast	Italy	European	40	15		25^b^	46	9		37^b^
Krippl 2004	breast	Austria	European	216	59	103	54	259	43	146	70
Fang 2005	NSCLC^c^	China	Asian	123	7	41	75	73	0	17	56
Cotignola 2008	melanoma	USA	European	129	21	69	39	853	148	428	277
Tu 2007	head/neck	Taiwan	Asian	59	12^a^		47	91	20^a^		71
Zhang 2004	ESCC^d^	China	Asian	59	1	26	32	72	0	20	52
Zhang 2004	GCA^e^	China	Asian	46	2	11	33	48	1	12	35
Smolarz 2003	ovarian	Poland	European	61	17	24	20	57	20	22	15
**MMP7 (−181) A/G**											
Hughes 2007	breast	London	European	49	17	20	12	81	30	39	12
Zhang 2005	ESCC^d^	China	Asian	68	61		7^b^	87	74		13^b^
Zhang 2005	GCA^e^	China	Asian	46	36		10^b^	63	56		7^b^
Zhang 2005	NSCLC^c^	China	Asian	123	101		22^b^	74	60		14^b^
Wu 2011	cervical	China	Asian	39	17	14	8	178	96	70	12
**MMP9 (−1562) C/T**											
Nasr 2007	head/neck	Tunisia	European	118	96	20	2	56	43	12	1
Woo 2006	colorectal	Korea	Asian	79	67	11	1	106	88	17	1
Xing 2007	colorectal	China	Asian	46	29		17^b^	87	71		16^b^
Hughes 2007	breast	London	European	43	35		8^b^	76	74		2^b^
Przybylowska2006	breast	Poland	European	141	83	56	2	129	90	38	1
Lei 2007	breast	Sweden	European	230	164	61	5	555	392	143	20
Wang 2005	NSCLC^c^	China	Asian	123	89		34^b^	74	59		15^b^
Matsumura 2005	gastric	Japan	Asian	63	44	16	3	114	89	22	3
Awakura 2006	renal	Japan	Asian	154	106		48^b^	25	20		5^b^
Park 2011	colorectal	Korea	Asian	132	107	24	1	201	163	37	1

a represents the number of AA+AB genotype,

b represents the number of BB+AB genotype (A represents the major allele, B represents the minor allele),

c NSCLC represents non-small cell lung carcinoma,

d ESCC represents esophageal squamous cell carcinoma.

e GCA represents gastric cardiac adenocarcinoma.

Different genotyping methods were used in these studies, including the classical polymerase chain reaction-restriction fragment length polymorphism (PCR-RFLP) in 21 of 33 studies [17]–[21], [23], [25]–[30], [39], [41]–[48], PCR-allele specific refractory mutation system analysis (ARMS) in 2 studies [7], [40], TaqMan assay in 4 studies [19], [22], [34], [37], PCR-sequencing in 6 studies [24], [31], [33], [35]–[36], [38], and PCR – fluorescent fragment analysis in 2 studies [28], [32].

### Quantitative data synthesis

#### MMP1 (−1607) 1G/2G

**Figure 2:**
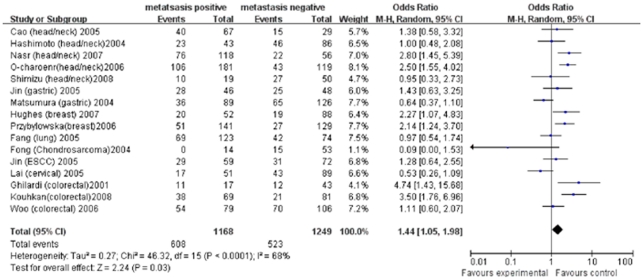
Forest plot of cancer metastasis risk associated with *MMP1 (−1607) 1G>2G* under the recessive model. A random-effects model was used. The *squares* and *horizontal line* represent the study-specific OR and 95% CI. The *diamond* represents the pooled results of OR and 95% CI.

Seventeen studies investigating *MMP1 (−1607) 1G/2G* and its association with cancer metastasis were identified [17]–[32], [46]. There were significant associations in overall comparison and subgroup analysis under the recessive model. *2G/2G* genotype increased the overall risk of metastasis (OR = 1.44, 95%CI = 1.05–1.98, *I* ^2^ = 68%, *p*<0.01) (Figure 2). Based on different cancer types, associations were also found in head/neck cancer (OR = 1.88, 95%CI = 1.39–2.53, *I* ^2^ = 48%, *p* = 0.1) and breast cancer (OR = 2.18, 95%CI = 1.40–3.40, *I* ^2^ = 0, *p* = 0.9). However, no significant association was found in colorectal, gastric and other cancers (including lung, cervical, esophageal cancer and chondrosarcoma) (Table 2). Compared to *1G/1G* genotype, genotype *2G/2G* or *1G/2G* showed no association with metastasis in overall analysis under the dominant model (OR = 1.24, 95%CI = 0.81–1.90, *I* ^2^ = 49%, *p* = 0.03). However, individuals with genotype *2G/2G* or *1G/2G* had higher risk of metastasis in breast cancer when stratified by cancer type (OR = 1.59, 95%CI = 1.02–2.48, *I* ^2^ = 0%, *p* = 0.69) (Table 2).

**Table 2:** Stratified analysis of MMP polymorphisms on cancer metastasis.

Variables	*N* a	Dominant genetic model			*N* a	Recessive genetic model		
		OR(95%CI)	*I^2^*	*P* b		OR(95%CI)	*I^2^*	*P* b
**MMP1 −1607**								
Tumor site								
head/neck	2^19–20^	1.53^c^(0.24–9.53)	76	0.04	5^17–18,20–22^	1.88(1.39–2.53)	48	0.1
colorectal	1^25^	1.91(0.36–10.09)	—	—	3^23–25^	2.45^c^(0.98–6.12)	75	0.02
gastric	2^26–27^	1.33(0.65–2.74)	54	0.14	2^26–27^	0.82(0.52–1.29)	61	0.11
breast	2^28–29^	1.59(1.02–2.48)	0	0.69	2^28–29^	2.18(1.40–3.40)	0	0.9
other	5^26,30–32.46^	0.89^c^(0.39–2.04)	62	0.03	4^26,30–32^	0.81(0.56–1.17)	47	0.13
Ethnicity								
Asian	7^19,25–27,30–32^	0.90(0.62–1.32)	41	0.1	10^17–18,21–22,25–27,30–32^	1.06^c^(0.76–1.48)	57	0.01
European	4^20,28–29,46^	1.86(1.25–2.78)	0	0.42	5^20,23–24,28–29^	2.68(1.96–3.66)	0	0.68
Total	11	1.24^c^(0.81–1.90)	49	0.03	15	1.44^c^(1.05–1.98)	68	<0.0001
**MMP2 −1306**								
Tumor site								
All	4^7,33–35^	0.61^c^(0.33–1.12)	83	0.0005	3^33–35^	1.17(0.75–1.83)	8	0.34
Ethnicity								
Asian	2^7,35^	0.31(0.18–0.54)	0	0.63	1^35^	0.95(0.21–4.35)	—	—
European	2^33–34^	1.03(0.81–1.32)	44	0.18	2^33–34^	1.19(0.75–1.90)	52	0.15
**MMP3 −1171**								
Tumor site								
breast	3^28,36–37^	0.56(0.39–0.79)	0	0.53	2^28,37^	0.80(0.55–1.17)	44	0.18
other	4^30,33,39–40^	0.99(0.67–1.46)	5	0.38	5^30,33,38–40^	0.80(0.62–1.03)	34	0.18
Ethnicity								
Asian	2^30,39^	0.21(0.04–1.02)	0	0.72	3^30,38–39^	0.64(0.44–0.92)	27	0.25
European	5^28,33,36–37,40^	0.76(0.58–0.99)	52	0.08	4^28,33,37,40^	0.89(0.69–1.16)	4	0.37
Total	7	0.72(0.56–0.93)	35	0.15	7	0.80(0.64–0.99)	25	0.23
**MMP7 −181**								
Total	3^28,45,47^	1.17(0.81–1.67)	0	0.45	2^28,47^	2.43(1.25–4.73)	0	0.33
**MMP9 −1562**								
Tumor site								
colorectal	3^25,41,48^	1.07(0.81–1.38)	43	0.15	2^25,48^	1.43(0.26–10.26)	0	0.95
breast	3^28–29,34^	1.23^c^(0.94–1.61)	77	0.01	2^29,34^	0.70(0.29–1.70)	0	0.4
other	4^20,42–44^	1.32(0.90–1.94)	0	0.45	2^20,43^	0.89(0.44–1.80)	0	0.76
Ethnicity								
Asian	6^25,41–44,48^	1.37(1.02–1.83)	5	0.38	3^25,43,48^	1.66(0.47–5.84)	0	0.98
European	4^20,28–29,34^	1.33^c^(0.74–2.36)	69	0.02	3^20,29,34^	0.72(0.31–1.65)	0	0.68
Total	10	1.25(1.03–1.51)	43	0.07	6	0.92(0.47–1.82)	0	0.85

a Number of comparisons.

b *P* value for *Q* test.

c Random effect model was used.

In the stratified analysis based on ethnicity of study population, there was a strong association between metastasis and *1G/2G* polymorphism in European populations under recessive and dominant models (dominant: OR = 1.86, 95%CI = 1.25–2.78; recessive: OR = 2.68, 95%CI = 1.96–3.66). However, this association was lost in Asian populations (Table 2).

#### MMP3 (−1171) 5A/6A

**Figure 3:**
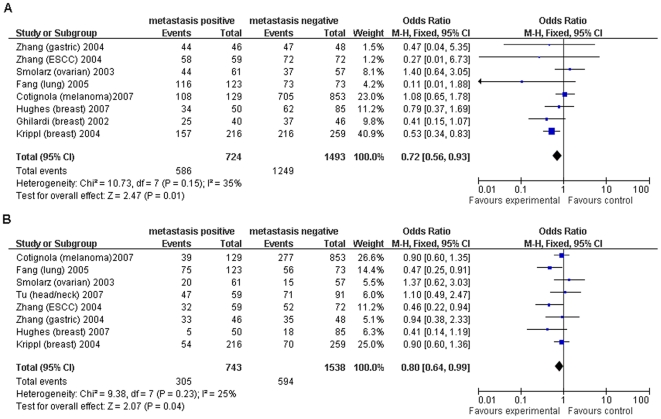
Forest plot of cancer metastasis risk associated with *MMP3 (−1171) 5A>6A*. A fixed-effects model was used. A indicates the result under the dominant model (*6A/6A+5A/6A* vs. *5A/5A*). B indicates the result under the recessive model (*6A/6A* vs. *5A/5A+5A/6A*). The *squares* and *horizontal line* represent the study-specific OR and 95% CI. The *diamond* represents the pooled results of OR and 95% CI.

Eight studies investigated *MMP3 (−1171) 5A/6A* and its association with cancer metastasis [28], [30], [33], [36]–[40]. Individuals with genotype *5A/6A or 6A/6A* had lower risk of metastasis under the two genetic models (dominant: OR = 0.72, 95%CI = 0.56–0.93; recessive: OR = 0.80, 95%CI = 0.64–0.99) (Figure 3). Stratified analysis by cancer type showed that this association was found in breast cancer under the dominant model (OR = 0.56, 95%CI = 0.39–0.79, *I* ^2^ = 0, *p* = 0.53). However, the association was lost under the recessive model.

In the stratified analysis by ethnicity, European individuals with genotype *6A/6A* or *5A/6A* had lower risk of metastasis under the dominant model (OR = 0.76, 95%CI = 0.58–0.99), whereas Asian individuals with genotype *6A/6A* had lower risk of metastasis under the recessive model (OR = 0.64, 95%CI = 0.44–0.92) (Table 2).

#### MMP9 (−1562) C/T

**Figure 4:**
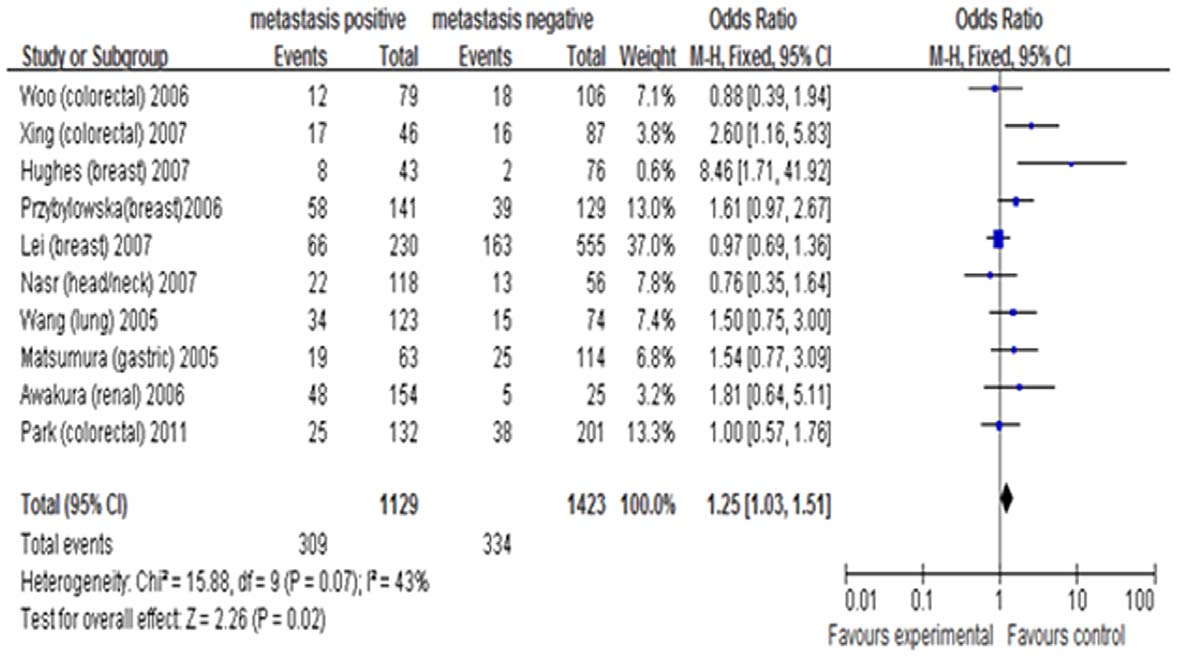
Forest plot of cancer metastasis risk associated with *MMP9 (−1562) C>T* under the dominant model. A fixed-effects model was used. The *squares* and *horizontal line* represent the study-specific OR and 95% CI. The *diamond* represents the pooled results of OR and 95% CI.

Ten studies evaluated *MMP 9(−1562) C/T* polymorphism and its association with cancer metastasis [20], [25], [28]–[29], [34], [41]–[44], [48]. Genotype *TT* or *CT* increased the overall risk of metastasis under the dominant model (OR = 1.25, 95%CI = 1.03–1.51, *I* ^2^ = 43%, *p* = 0.07) (Figure 4). However, no association was found between genotype *TT* and metastasis under the recessive model. In stratified analysis by cancer type, there was no significant association under the two genetic models. Based on the ethnicity of study population, association was found in Asian populations only under the dominant model (OR = 1.37, 95%CI = 1.02–1.83, *I* ^2^ = 5%, *p* = 0.38), while no association was found under the recessive model (Table 2).

#### MMP2 (−1306) C/T and MMP7 (−181) A/G

**Figure 5:**
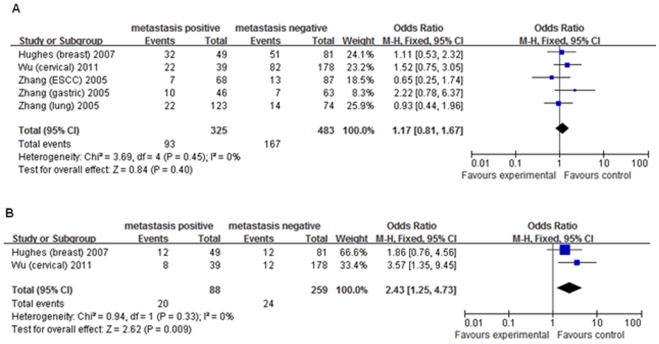
Forest plot of cancer metastasis risk associated with *MMP7 (−181) A>G*. A fixed-effects model was used. A indicates the result under the dominant model (*GG+AG* vs. *AA*). B indicates the result under the recessive model (*GG* vs. *AG+AA*). The *squares* and *horizontal line* represent the study-specific OR and 95% CI. The *diamond* represents the pooled results of OR and 95% CI.

Four studies evaluated *MMP2 (−1306) C/T* and its association with cancer metastasis [7], [33]–[35], and only three evaluated the association between *MMP7 (−181) A/G* and metastasis [28], [45], [47]. For *MMP7 (−181)*, there was an association between *GG* genotype and risk of metastasis under the recessive model (OR = 2.43, 95%CI = 1.25–4.73), however, no association was found under the dominant model (Figure 5). Our analysis did not provide any statistical evidence of association between *MMP2* polymorphism and risk of metastasis (Table 2).

### Heterogeneity analysis

For *MMP1 (−1607)1G/2G*, significant heterogeneity was found in overall comparisons under the two genetic models (dominant: *I^2^* = 49%, *p* = 0.03; recessive: *I^2^* = 68%, *p*<0.01). The *I^2^* decreased obviously and *p* value exceeded 0.05 after excluding the study of Lai [32] under the dominant model (*I^2^* = 26%, *p* = 0.20), indicating that this study was the major source of heterogeneity. The significance of pooled ORs and 95%CI under the dominant model in both overall comparison and subgroup analysis was not influenced by omitting Lai's study. Heterogeneity under the recessive model was still significant after excluding Lai's study (*I^2^* = 63%, *p* = 0.0005), but it was eliminated after excluding four studies [24], [27], [31]–[32] (*I^2^* = 44%, *p* = 0.05). The significance of pooled ORs under the recessive model was also not influenced by omitting those four studies. In the present study, most genotype data were based on the time of diagnosis except for the studies [21]–[22] on the time of follow-up. Therefore, sensitivity analysis was performed by omitting the two studies. The overall result was not influenced (OR = 1.63, 95%CI = 1.22–2.18).

The results of meta-regression for *MMP1 (−1607) 1G/2G* indicated that cancer site and ethnicity of study population independently contributed to the heterogeneity observed under dominant and recessive models (data not shown). Effects of cancer type on heterogeneity were significant under dominant and recessive models (dominant: *p* = 0.084<0.1, recessive: *p* = 0.047<0.1). Genotyping methods, sample size, and publication year were not statistically associated with heterogeneity.

For *MMP2 (−1306) C/T*, heterogeneity between studies was statistically significant under the dominant model (*I* ^2^ = 83%, *p*<0.01). The heterogeneity was eliminated after excluding two studies [7], [35] (*I^2^* = 44%, *p* = 0.18). The significance of pooled ORs and 95%CI was not influenced by omitting the two studies.

Genotype data of study [36] for *MMP3 (−1171) 5A/6A* were based on the time of follow-up. As selective bias for the result might exist, we performed sensitivity analysis by omitting this study. The significant association remained unchanged (OR = 0.76, 95%CI = 0.58–0.99).

For *MMP9 (−1562) C/T*, heterogeneity was statistically significant in the subgroup analysis based on cancer type and ethnicity of study population under the dominant model (Table 2). The *I^2^* decreased and *p* value exceeded 0.05 after excluding the study of Hughes [28], suggesting that this study was the major source of heterogeneity. The significance of pooled ORs and 95%CI was not influenced by omitting Hughes' study.

### Publication bias analysis

Publication bias was assessed by performing funnel plot and Egger's regression test under the dominant and recessive models. If the number of included studies was small, it is unnecessary to perform publication bias analysis. After combining all the cancer types, a little asymmetry was observed for *MMP1 (−1607)1G/2G*, but the results of Egger's regression test suggested no evidence for publication bias (dominant: t = −0.63, *p* = 0.54; recessive: t = −0.66, *p* = 0.517). For *MMP3 (−1171) 5A/6A* and *MMP9 (−1562) C/T*, funnel plots were symmetrical and the Egger's test for both models showed no significance, suggesting little evidence of publication bias.

## Discussion

In our comprehensive meta-analysis, *MMP1 (−1607)1G/2G*, *MMP7 (−181) A/G* and *MMP9 (−1562) C/T* were shown to increase the risk of cancer metastasis, whereas *MMP3 (−1171) 5A/6A* was protective in metastasis. Meanwhile, there was no association between *MMP2 (−1306) C/T* and metastasis.

MMP1 is implicated in cancer susceptibility and metastasis in a variety of cancers. A single nucleotide polymorphism at −1607 bp in the *MMP1* promoter is described in Rutter's study [8]. This promoter region is characterized by a *1G/2G* polymorphism, where *2G* allele creates an Ets-binding site and increases the transcriptional activity compared to *1G* allele. In our analysis, *2G/2G* genotype increased the risk of metastasis under the recessive model, whereas no association was found in the dominant model. The result demonstrates that homozygous *2G* has a stronger effect on an individual's phenotype than heterozygous *2G*. Therefore, individuals with *2G/2G* genotype have a higher risk of metastasis than those with *1G/2G* genotype. When stratified by cancer types, this association was found in head/neck cancer and breast cancer under the recessive model. Results for different cancer types were inconsistent, which might be caused by the different microenvironments and mechanisms in different cancer types. When we conducted a subgroup analysis based on ethnicity, significant associations were only found in the European populations under the two genetic models. In our analysis, populations selected in the two studies on breast cancer were all European, which might cause selection bias. Therefore, we could not conclude that European populations with this polymorphism have a higher risk of metastasis than Asian populations.

The promoter region of *MMP3* gene contains an adenosine insertion/deletion polymorphism located at −1171 bp relative to the transcriptional start site, where one allele has five adenosines and the other has six adenosines. It is implicated that the transcriptional activity of *MMP3* in individuals with a *5A* allele is twice that in individuals with a *6A* allele [9]. In overall comparison, *5A/6A* polymorphism had a protective role in metastasis under the two genetic models. The result in dominant model was more evident than that in recessive model, and it was demonstrated that the heterozygous *6A* had a stronger effect on an individual's phenotype than homozygous *6A*. Therefore, individuals with *5A/6A* genotype had an apparent protective role in metastasis compared to those with *6A/6A* genotype. When stratified by cancer type, this protective role was only found in breast cancer under dominant model. As mentioned above, this result may be caused by different microenvironments in different cancers. In the subgroup analysis, *5A/6A* polymorphism decreased the risk of metastasis in European populations under dominant model, and this protective role was found in Asian populations under recessive model. As the populations in the three studies on breast cancer were all European, selection bias may exist and the final result may be influenced. The association between ethnicity and metastasis remains uncertain, waiting to be analyzed by further studies using larger sample size.

MMP9 is the most complex member of MMPs, which plays an important role in metastasis. The *C* to *T* substitution in the promoter region of *MMP9* gene has a higher transcriptional activity of the T-allelic promoter, which might be caused by DNA-protein interaction abolishment by the *C* to *T* substitution at this polymorphism site [10]. Significant association between this polymorphism and metastasis was only found in dominant model. This result shows that individuals with *CT* genotype have a higher risk of metastasis than those with *TT* genotype. When analyzed based on ethnicity of study population, Asian populations with genotype *TT* or *CT* had a higher risk of metastasis, in contrast, no association was found in European populations. This result demonstrates that Asian populations with this polymorphism might be susceptible to metastasis compared to Europeans under dominant model. In our analysis, no significant heterogeneity was found in overall comparisons under the two genetic models.

An *A* to *G* transition at −181 base pair position upstream of the transcription start site of *MMP7* gene has been reported. The *G* allele has greater basal transcriptional activity than *A* allele in vitro experiment [12]. Our results demonstrate that individuals with *GG* genotype could increase the risk of metastasis, and this result is consistent with the above hypothesis. The promoter region of *MMP2* has been shown to contain several cis-acting regulatory elements, and a *−1306 C to T* transition interrupts Sp1-binding site and diminishes the promoter activity [11]. For *MMP2 (−1306) C/T*, no statistical association and significant heterogeneity were found in the overall comparison and subgroup analysis. Because there are only four studies for *MMP2*, the negative results do not mean that there was no association with metastasis.

Results for different MMP polymorphisms in metastasis are inconsistent, which can be explained by several reasons. First, the study population in each report comes from different areas and races. Different genetic backgrounds and environmental factors could influence the results. Second, the small sample size in some studies might influence the overall effect. It is necessary to gather studies with larger sample sizes to decrease the possibility of false positive and negative. Third, different MMP regulation mechanisms and microenvironments in different cancers may explain why MMP polymorphisms play different roles in cancer metastasis. Fourth, some cases are gynaecological cancers. The development and metastasis of gynaecological cancers could be influenced by some environmental factors and other factors including oestrogen, pregnancy and coitus.

Heterogeneity is an important problem when interpreting the results of our meta-analysis. In this study, significant heterogeneity was found in three of the five polymorphisms. For these polymorphisms, the heterogeneity disappeared after excluding several studies. Results of meta-regression demonstrate that cancer type and ethnicity of the studied population are the major source of the heterogeneity. Because the genotype data of studies [21]–[22], [36] were based on the time of follow-up, sensitivity analysis was done by omitting these three studies, and the results were not influenced by omitting them. Therefore, the three studies were included in our studies.

There are some limitations in our analysis. First, although we collected all the eligible studies, the sample size of the included studies was not large enough, which could increase the likehood of type I and type II errors. Therefore, there was a lack of statistical power to better evaluate the association between MMP polymorphisms and metastasis, especially in subgroup analysis. Second, we showed the results by combining all cancers, however, the results in subgroup analysis were more meaningful. We only analyzed the data based on different cancer types and ethnicity of the studied population due to the limited data. Third, gene-gene and gene-environment interactions were not analyzed. It is possible that specific environmental and lifestyle factors may alter those associations between gene polymorphisms and metastasis. Therefore, it is necessary to evaluate the roles of some special environment factors and lifestyles such as diet, alcohol consumption and smoking status in metastasis. Fourth, although the funnel plot and Egger's test did not show any publication bias, the influence of bias in the present analysis could not be completely excluded. For example, studies with positive results are more easily published than those with negative results, and only studies published in English are included. Finally, as we only focused on the associations of MMP polymorphisms with cancer metastasis in the present study, the significance was limited. To ensure the validity and reliability of the conclusions, it is important to perform a meta-analysis on the associations between metastasis positive cases vs. healthy controls and negative cases vs. healthy controls in the future study.

In conclusion, the results in our meta-analysis demonstrate that the polymorphisms of *MMP1, 3, 7* and *9* have significant associations with the risk of metastasis, although some results are limited by the small number of studies. However, no significant association exists between *MMP2 (−1306) C/T* and metastasis. This polymorphism may not be the major risk of metastasis. Further studies with large sample size are needed to evaluate its association with metastasis.

## Supporting Information

Table S1 **The criteria of quality evaluation for every included study.**
(DOC)

Table S2 **The results of quality evaluation for every included study.**
(XLS)

Table S3 **Main characteristics of all studies included in the meta-analysis.**
(XLS)
