# Supplementary material for: Association between Polymorphisms in the Promoter Regions of Matrix Metalloproteinases (MMPs) and Risk of Cancer Metastasis: A Meta-Analysis
Source: PLoS One. 2012 Feb 14;7(2):e31251. doi: 10.1371/journal.pone.0031251 (PMC3279370; doi:10.1371/journal.pone.0031251)
Supplement: Table S1 — The criteria of quality evaluation for every included study. (DOC) [file pone.0031251.s001.doc]

**Table S1 The criteria of quality evaluation for every included study.**

1. **Is the Case Definition Adequate?**
2. Yes, with independent validation (1 score)
3. Yes, but record linkage or self-report with no reference to primary record (0 score)
4. No description (0 score)
5. **Representativeness of the Cases**

a) Consecutive or obviously representative series of cases (1 score)

b) Potential for selection biases or not stated (0 score).

1. **Selection of Controls**
2. Community controls or hospital-based controls (1score)
3. Hospital controls, but derived from a hospitalised population (0 score).
4. No description (0 score).
5. **Definition of Controls**

a) No history of disease (1score)

b) No description of source (0 score)

1. **Comparability of Cases and Controls on the Basis of the Design or Analysis**

a) Study controls for the main confounding factors (1 score)

b) Study controls for any confounding factors (1 score)

c) No control (0 score)

1. **Ascertainment of Exposure**

a) Definite records (such as surgical records) or structured interview where blind to case/control status (1 score)

b) Written self report or medical record only (0 score)

1. No description (0 score)
2. **Non-Response Rate**
3. Consistency for cases and control (1 score)
4. Description for non respondent (0 score)
5. Inconsistency or No description (0 score)
6. **Method of determination for cases and controls**
7. Consistency (1 score)
8. Inconsistency (0 score)

**Note:**

If the total scores are over 6, the quality of the paper is good, otherwise, the quality is poor (score: 0-5).
